# Discussing end of life wishes – the impact of community interventions?

**DOI:** 10.1186/s12904-019-0407-8

**Published:** 2019-03-07

**Authors:** Katharine Abba, Mari Lloyd-Williams, Siobhan Horton

**Affiliations:** 1 Department of Public Health and Policy, University of Liverpool, 3rd floor, Whelan Building, Liverpool, L69 3GB UK; 2 Academic Palliative and Supportive Care Studies Group, Department of Health Services Research, Ist floor, Block B, Waterhouse Building, Liverpool, L69 3BX UK; 3 End of Life Partnership, Cheshire, Winterley Grange, Unit 8, Wheelock Heath Business Court, Alsager Road, Winterely, Sandbach, Cheshire CW11 4RQ UK

**Keywords:** End of life, Community development, Wills, Funerals, Dying, Death, Bereavement, Grieving, Health promotion

## Abstract

**Background:**

Many people do not discuss end of life preferences with those closest to them, although this can be beneficial to the individual and wider population. This study evaluated a community intervention to promote end of life preparation and discussion among people who are currently well.

**Methods:**

A series of presentations and workshops (the intervention) were delivered to community groups and people working within health and social care. Participants were invited to complete a three-stage follow-up survey at Baseline, Post intervention and at three months' follow-up.

**Results:**

Baseline questionnaires were completed by 498 individuals. Overall, 51% reported talking with close family or friends about their end of life care and 58% reported talking about what they would like to happen after their death. There was a significant positive relationship between increasing age group and having talked about end of life wishes. The majority of participants were already comfortable in talking about end of life (overall mean score 8.28/10). Post intervention, 73% stated that they planned to take action including 61% who planned a specific conversation and 55% who planned another action. At follow-up 64% reported that they had taken some action due to the intervention, including 43% who had talked about their own end of life preferences and 39% who had taken some other action.

**Conclusions:**

Well-designed community-based interventions can be successful in prompting people to consider and discuss their end of life preferences.

**Electronic supplementary material:**

The online version of this article (10.1186/s12904-019-0407-8) contains supplementary material, which is available to authorized users.

## Background

Each year in England and Wales approximately 1% of the population die [1] and 5% are directly affected through caring and bereavement [2]. Dying, caring, or grieving can be stressful, and leave individuals feeling isolated and unsupported [3, 4]. Public health approaches, such as Health Promoting Palliative Care [5] and Compassionate Cities [6] have been developed on the assumption that that communities, while wishing to support their members, might feel uncomfortable talking about death and dying. As a result, people can be unprepared for death and unable to support others during dying and bereavement.

There is evidence that discussing end of life wishes can be beneficial. In studies undertaken in the USA, those who completed an Advance Directive were more likely to receive the end of life care they desired if they had also discussed their wishes [7], and where power of attorney was given to relatives, relatives found it easier to make decisions if issues had been discussed [8]. There are obvious benefits to having these discussions while still well. Dying trajectories do not always include a period when death is expected and preparations might be made: around 15% of deaths are sudden [9], people may be unaware that a condition is life-limiting, and many conditions have an unpredictable trajectory. People with newly-diagnosed life-limiting illness often find it difficult to talk about their end of life preferences [10], and others may be preoccupied with day to day living and survival [11, 12], avoid thinking about death in order to better enjoy the present [13], or feel too unwell to make plans [14].

Despite the potential benefits, many people do not talk about their end of life preferences. In a recent UK population survey [15], only 50% reported telling anybody whether they would like to be buried or cremated and only 37% had made a will. This varied by age group, with a greater proportion of older compared with younger participants having made a will or talked about their funeral wishes. Other surveys have reported similar findings [16–18].

There is currently limited evidence on effective public health interventions. Suggested initiatives include education to help communities prepare for death and activities to ‘normalise’ death as something that can be talked about [10, 19]. A recent systematic literature review identified five relevant published studies [20] and concluded that, in the right circumstances, people often appreciate the opportunity to discuss end of life issues.

In 2005, The International Work Group on Death, Dying and Bereavement [21] recommended a combined approach from Public Health and End of Life Care providers to the normalisation of death, dying and loss within society at large. The Cheshire Living Well Dying Well (CLWDW) Public Health Programme, led by St Luke’s Hospice, Cheshire was established to support these aims within the county of Cheshire, England. In 2011, a dedicated public health lead was appointed, and in 2012, a public health worker was appointed to assist with the design and delivery the programme.

This study evaluated the impact of a CLWDW community public health intervention designed to help normalise death and promote preparation and discussion of end of life preferences within the local community.

## Aims

To examine the impact of the CLWDW community public health interventions in the short and medium term.

## Methods

### The intervention

Two different types of intervention were evaluated; ‘Awareness-Raising’ presentations (*N* = 40) and ‘How to’ workshops (*n* = 21), delivered between February 2013 and April 2014.

‘Awareness-Raising’ presentations aimed to raise awareness of the benefits of planning for end of life and talking about plans and preferences. Events were 60 to 90 min long and delivered free of charge to community groups (*N* = 37); the general public (*N* = 2); and to health and social care staff, in this case branded as ‘Making the Professional Personal’ (*N* = 1). Presentations delivered to community groups were advertised to group members as ‘visiting speakers’ and held at the same time and location as the groups usually met. Presentations for the general public were openly advertised, and presentations aimed at health and social care staff were advertised through local employers.

Presentations were facilitated by the public health worker, supported by volunteers. They included PowerPoint-aided talks, group discussions, and showing of three films. ‘Cheshire Bill United’ was adapted from ‘Bill’s Story’ produced by the Milford Care Centre in Ireland [22], an animation of community coming together to support a man and his family as he is diagnosed with a terminal illness and dies. The ‘Circle of Life’, was an animated montage produced by the CLWDW Public Health Lead, illustrating that death could be made easier, especially for those left behind. ‘Dying for a Laugh’ was adapted from a film produced by the National Dying Matters Coalition [23], comprising clips of comedians talking about death. After the event, the facilitators remained to answer questions and talk with individuals as needed.

‘How to’ workshops aimed to increase confidence and equip participants with tools to facilitate conversations with people close to them about end of life plans and preferences. Workshops were delivered to community groups (*N* = 10) and to the general public (*N* = 5) as ‘Dying to Talk’. They were presented to health and social care staff as ‘How to: Making the professional personal’ (*N* = 6). The five workshops for the general public were advertised through awareness-raising events as a ‘follow-up’. Workshops aimed at health and social care staff were advertised through employers; there was no requirement for attendees to have previously attended an ‘awareness-raising’ session.

Workshops were around 150 to 180 min long, free of charge and delivered jointly by the public health worker and another facilitator. They began with discussion of the benefits of talking about end of life preferences and what prevents conversations, followed by presentation of ideas that might help, using symbols as visual aids. Ideas presented included: ‘talking upstream’ (discussing end of life when it appeared a long way off); ‘planning’ (planning what to say); ‘practice’ (practicing what to say); ‘triggers’ (finding a suitable trigger for the conversation, such as a story in a soap opera); ‘listening’ (listening carefully to what the other person is saying); and ‘starting’ (starting by telling the other person your own end of life wishes).

One or two video vignettes, produced by CLWDW, depicted scenarios of a daughter and mother, or a wife and husband; one wanting to talk about the other’s end of life preferences and the other being reluctant to have the conversation. Both scenarios included examples of ‘poor communication’ and ‘good communication’. Discussions followed, ending with a summary of the learning and a short presentation about wills; power of attorney; advance care planning; funeral plans; letters of wishes; emotional wills and bucket lists. Information packs were provided to take away.

### The research

The research comprised three linked questionnaires: ‘Baseline’ administered immediately prior to the intervention; ‘Post’ administered immediately afterwards; and ‘Follow-up’ administered three months later (Additional files 1, 2 and 3). All included fixed and free text response questions. The questionnaires were developed by the main researcher (KA) in consultation with the wider research team and public health worker, and piloted at a pilot Awareness-Raising event attended mainly by CLWDW volunteers. They included questions that were purposely similar to questions included in the Dying Matters 2012 survey [15] to allow comparison with a representative national sample and ‘new’ questions designed to measure outcomes relating to the objectives of the events. At baseline, demographic information, including age group, sex and socio-economic role, was gathered to monitor the reach of the intervention and determine whether there were any significant differences in needs or response between groups with different characteristics. Age group was collected in preference to exact age as we felt this might be more acceptable to the participants; the age bands used mirrored those used in the Dying Matters surveys [15]. Full postcode was requested, to facilitate linkage with public datasets. The ‘Post’ questionnaire requested permission to contact for follow-up and postal address.

The ‘Baseline’ and ‘Post’ questionnaires were printed together as a single booklet, each with a unique code. The public health worker gave a questionnaire booklet to attendees as they arrived at event, and reminded them to complete the ‘Baseline’ questionnaire before the event started and the ‘Post’ questionnaire before leaving. Where permission and postal address were available, the researcher sent ‘Follow-up’ questionnaires approximately three months later, with pre-paid return envelopes and personally signed thank you notes. Each questionnaire was printed with the unique code assigned at Baseline. Where questionnaires were not returned within two weeks, one reminder was sent.

As far as possible, all persons who attended events and expressed a willingness to participate were included in the study. Clipboards and pens were provided, and assistance given as needed. Respondents were recruited at 61 of the 64 events taking place during the study period, including 40 ‘Awareness Raising’ presentations and 21 ‘How To’ workshops, attended by a total of 676 people. Three events were not included; two due to insufficient time, one because the facilitator was aware that two attendees had learning disabilities and wanted to avoid drawing attention to that. Because the majority of events were delivered to established groups during their usual meeting times, it was not practical to collect demographic information on the participants who declined to attend or take part.

Data was entered manually into a Microsoft Access 2007 database, using an electronic form with appropriate validation checks. Data was then linked by postcode to Census 2011 lower level super output area (LSOA) [24] and LSOA linked to rank of deprivation of LSOA according 2010 English Index of Multiple Deprivation (IMD 2010) [25]. The full dataset was then exported into Microsoft Excel 2007 and SPSS Statistics 2 for analysis.

For analysis, ratings scores were treated as continuous data. An independent samples t-test was used to compare group mean scores, and a related samples t-test was used to compare mean scores before and after the intervention. For categorical data, a chi-squared test and a chi-squared test for trend were used to compare proportions of groups of respondents. For age groups, the original ten-year age-bands were collapsed into larger bands according to visual trends observed in the distribution of the data. An exact test for paired data was used for comparing proportions before and after the intervention. Two-sided significance tests were used, except where change in scores or proportions could only take place in one direction. Where appropriate, logistic regression models were constructed and run in order to measure the independent effects of respondent variables on binary outcomes. For all tests, a conventional criterion of statistical significance (*P* < 0.05) was used. Responses to ‘open’ questions were analysed thematically by a single researcher, using an inductive approach; the number of responses within each theme were then counted, and the findings presented numerically.

## Results

### Response rate

**Fig. 1:**
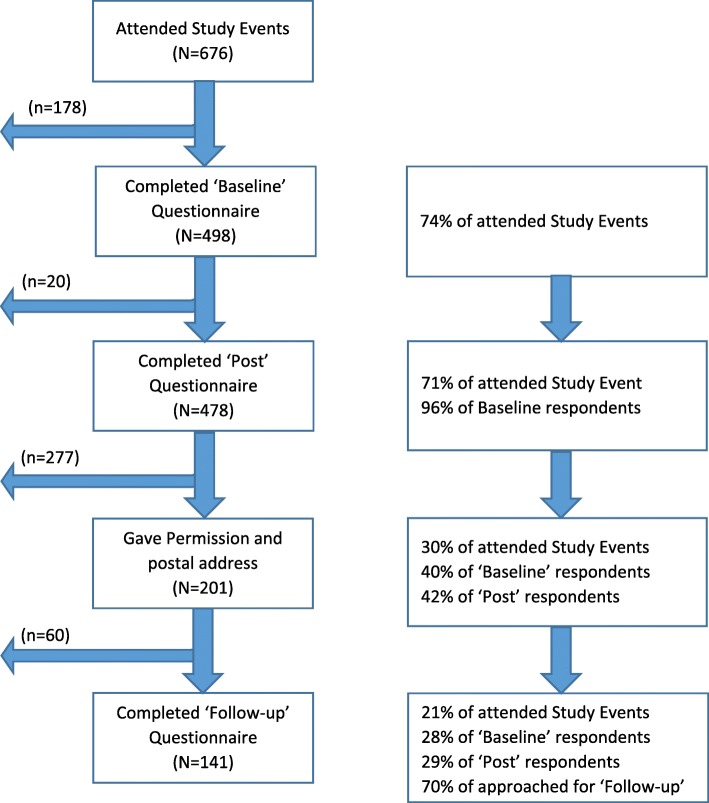
Number of participants at each stage of the study

Sixty-one intervention events were attended by 676 people; median attendance nine persons per event. Figure 1 shows the numbers and percentages participating in each stage of the study.

### Baseline and post-event

#### Demographics

The baseline sample included 498 individuals (74% of attendees), of whom 377 (76%) were female. Age groups ranged from under 25 to over 85; including 99 (20%) aged under 45; 327 (66%) aged 45 to 74; and 75 (15%) aged 75 or older. Respondents attending ‘How to’ workshops were younger on average than those attending ‘Awareness-raising’ presentations (29% v 54% over the age of 65).

The most frequently reported socio-economic role was retired (233, 47%) followed by working part-time (120, 24%); and working full-time (93, 19%). Around a quarter (116, 23%) lived alone. Others reported living with a spouse or partner (*n* = 299, 60%); with a spouse or partner plus other family (26, 5%); or with family members other than a spouse or partner (43, 9%).

A valid English postcode was provided by 441 respondents, of whom 218 (49%) resided in LOSAs within the least deprived quintile in England and 14 (3%) resided in the most deprived quintile. This reflected the socio-economic characteristics of the area, which had relatively low levels of deprivation.

#### Preparations for end of life

At baseline, 68% respondents reported having a will, and a further 24% indicated they were thinking of making a will. There was considerable variation by age group, with 0% of under 35 s and 95% of over 75 s reporting having a will (chi-square test for trend *p* = < 0.001) (Table 1). There was also variation by neighbourhood deprivation, from 29% in the most deprived quintile within England to 78% within the least deprived quintile (chi-squared test for trend *p* = < 0.001), which was significant even when age group was taken into account (*P* = 0.009).

**Table 1:** Number and percentage of respondents who reported they had made a will or were considering making a will, and had had conversations with people close to them about end of life-related subjects, by age group

*Survey item*	*Age Group*							
	Under 35 (*n* = 48)	35 to 44 (*n* = 51)	45 to 54 (*N* = 70)	55 to 64 (*N* = 104)	65 to 74 (*N* = 147)	Over 75 (*n* = 76)	All ages (*n* = 496)	Chi-square test for trend
Already has a will	0 (0%)	17 (33%)	35 (50%)	79 (67%)	132 (90%)	73 (95%)	335 (68%)	*P* = < 0.001
Considering making a will	27 (58%)	29 (56%)	29 (41%)	20 (19%)	12 (8%)	2 (3%)	119 (24%)	*P* = < 0.001
No will and not considering making one	21 (42%)	5 (10%)	6 (9%)	5 (5%)	3 (2%)	2 (3%)	42 (8%)	*P* = < 0.001
Discussed own wishes for end of life care	13 (27%)	20 (39%)	37 (52%)	57 (55%)	81 (55%)	44 (60%)	252/495(51%)	*P* = < 0.001
Discussed own wishes for after death	15 (31%)	23 (45%)	46 (65%)	66 (64%)	83 (57%)	52 (74%)	285/489(58%)	*P* = < 0.001
Discussed another’s wishes for end of life care	23 (48%)	29 (57%)	43 (61%)	60 (57%)	78 (53%)	30 (42%)	263/492(54%)	NS
Discussed another’s wishes for after death	22 (46%)	27 (54%)	48 (69%)	59 (57%)	73 (51%)	25 (36%)	254/484(53%)	NS
Comforted somebody who has been bereaved	45 (94%)	38 (75%)	63 (89%)	95 (91%)	116 (79%)	58 (81%)	415/492(84%)	NS

Fifty-one percent reported discussing their own end of life care wishes with a close friend or family member; 285 (58%) reported discussing what they wished to happen after their death; and 332 (67%) reported discussing either of these two subjects. Similar proportions reported discussing another person’s end of life care wishes (263, 54%); another’s wishes for after they have died (254, 53%), or either of these (300, 60%).

Overall, 386 (78%) reported having some discussions with friends or family about either their own or the other’s end of life preferences. A larger proportion (415, 83%) reported supporting or comforting somebody who had been bereaved.

The proportion reporting talking about their own end of life preferences varied by age group, with older age groups more frequently reporting these discussions (*P* = < 0.001) (Table 1). There were no differences by sex or neighbourhood deprivation.

### How comfortable were respondents in talking about end of life preferences and bereavement?

Respondents were asked to indicate how comfortable they were, on a scale of 1 to 10, in talking about end of life preferences and bereavement. For each survey item, the most frequent response was 10 (completely comfortable) and the mean rating was around 8. The ranges and means are shown by age group in Table 2. There were statistically significant differences between respondents aged under 65 and those age 65 or older, and between those who had previously discussed the subject and those who had not. There were no significant differences by sex or neighbourhood deprivation.

**Table 2:** Distribution of score categories, and mean scores for how comfortable baseline survey respondents stated they were about talking with close friends and family about different aspects of end of life preferences, or comforting a person who has been bereaved

*Survey item*	*Survey Response*						
	Very uncomfortable (1 or 2)	Quite uncomfortable (3 or 4)		Neutral (5 or 6)	Quite comfortable (7 or 8)	Very comfortable (9 or 10)	
Talking about own end of life care	8 (2%)	29 (6%)		58 (12%)	117 (24%)	283 (57%)	
Talking about own wishes for after death	8 (2%)	29 (6%)		56 (12%)	113 (23%)	283 (58%)	
Talking about another’s end of life care	9 (2%)	34 (7%)		80 (17%)	124 (26%)	236 (49%)	
Talking about another’s wishes for after death	9 (2%)	32 (6%)		80 (17%)	126 (26%)	236 (49%)	
Comforting somebody who is bereaved	4 (1%)	22 (5%)		54 (11%)	143 (29%)	266 (54%)	
*Survey item - Mean score (95% CI)*	*Age Group*						
	Under 35	35 to 44	45 to 54	55 to 64	65 to 74	Over 75	All ages
Talking about own end of life care	7.77 (7.07 to 8.47)	7.82 (7.15 to 8.49)	8.32 (7.83 to 8.82)	8.26 (7.83 to 8.69)	8.63 (8.32 to 8.95)	8.59 (8.04 to 9.14)	8.28 (8.08 to 8.48)
Talking about own wishes for after death	7.65 (6.93 to 8.36)	7.68 (6.99 to 8.37)	8.49 (8.01 to 8.96)	8.26 (7.83 to 8.68)	8.47 (8.13 to 8.81)	8.84 (8.37 to 9.32)	8.28 (8.08 to 8.47)
Talking about another’s end of life care	7.15 (6.39 to 7.90)	7.40 (6.67 to 8.04)	7.79 (7.22 to 8.37)	7.85 (7.40 to 8.30)	8.41 (8.11 to 8.71)	8.41 (7.85 to 8.96)	7.93 (7.73 to 8.13)
Talking about another’s wishes for after death	6.92 (6.15 to 7.68)	7.48 (6.81 to 8.15)	7.84 (7.28 to 8.40)	8.05 (7.64 to 8.46)	8.30 (7.99 to 8.61)	8.55 (8.00 to 9.09)	7.95 (7.76 to 8.15)
Comforting somebody who has been bereaved	8.02 (7.42 to 8.62)	7.68 (7.06 to 8.30)	8.18 (7.71 to 8.65)	8.22 (7.84 to 8.59)	8.62 (8.32 to 8.92)	8.94 (8.55 to 9.33)	8.33 (8.15 to 8.51)
*Survey item - Mean score difference*	Under 65/65 and over			Previously talked about the subject/not talked about it			
Talking about own end of life care	0.45	(*p* = 0.02)		1.18	(*p* = < 0.001)		
Talking about own wishes for after death	0.48	(*p* = 0.019)		1.88	(*p* = < 0.001)		
Talking about another’s end of life care	0.72	(*p* = < 0.001)		1.31	(*p* = < 0.001)		
Talking about another’s wishes for after death	0.66	(*p* = 0.001)		1.47	(*p* = < 0.001)		
Comforting somebody who has been bereaved	0.66	(*p* = < 0.001)		1.75	(*p* = < 0.001)		

### How relevant were the events to those who attended them?

Respondents rated the personal relevance of events on a scale of one (completely irrelevant) to five (completely relevant). For both types of event, the most frequent response was five. The mean rating score was 4.15 (95% CI 4.04 to 4.26) for ‘Awareness Raising’ presentations and 4.23 (95% CI 4.08 to 4.37) for ‘How to’ workshops.

Events attended specifically by health and social care staff were rated as more relevant than those attended by community groups (4.67, 95% CI 4.28 to 5.00 v 4.13, 95% CI 4.02 to 4.25). Respondents aged 45 to 74 (4.32, 95% CI 4.23 to 4.42) reported events to be more relevant than those aged under 45 (3.88, 95% CI 3.65 to 4.11) or over 75 (3.91, 95% CI 3.65 to 4.16). There were no significant differences by sex or level of neighbourhood deprivation.

Participants were also asked for general comments about the event. Most comments were positive, including praise for the content, the opportunities for discussion, the sensitive facilitation, and the use of humour. Some expressed surprise that the event was not ‘morbid’.

### Changes in intentions post-event

There was a significant increase post-intervention in the proportion of people without a will who indicated they were thinking of making a will (74% v 90%, *P* = < 0.0001).

Respondents were asked to indicate whether, due to intervention, they intended to have a specific conversation, or to take another action. Overall, 70% attending Awareness raising presentations and 79% attending ‘How to’ workshops stated their intention to take action (Table 3).

**Table 3:** Percentage of ‘Post’ survey respondents who answered ‘yes’ to questions about whether they planned any specific conversations or other actions relating to the event they had attended

	Awareness (*n* = 326)	How to (*n* = 172)	Total (*n* = 498)
Are you planning any specific questions with family or friends because of anything you have heard today?	187 (57%)	119 (69%)	306 (61%)
Did the presentation inspire you to do anything else or make any other changes in your life?	168 (52%)	106 (62%)	274 (55%)
*Answered ‘yes’ to either question above*	***227 (70%)***	***136 (79%)***	***363 (73%)***

The most frequently mentioned conversations included own end of life or funeral wishes (*n* = 75); mutual wishes with somebody close (*n* = 75), and another’s wishes (*n* = 46). End of life care or resuscitation was mentioned by 22. Other subjects included organising financial arrangements for after death (*n* = 27), telling people where important documents were kept (*n* = 7), or persuading other family members to talk about their end of life wishes or make preparations such as a will.

There were four main themes relating to actions other than specific conversations. The first was practical matters such as making a will (*n* = 24); changing an existing will (*n* = 23); stating or updating written wishes (*n* = 24) planning for or updating plans for a funeral (*n* = 24); tidying up or decluttering (*n* = 7), starting a life book or ICE file (*n* = 5), obtaining a donor card (*n* = 3), or making plans for own end of life care (*n* = 3). The second was personal and emotional plans, including writing an emotional will or letter to be opened after death (*n* = 17), starting a journal or recording family history (*n* = 14); or writing a bucket list (*n* = 20). The third was changing general approaches to talking about death, including talking more or being more open (*n* = 15), being more confident (*n* = 10), listening better (*n* = 9) and being more supportive of people who were bereaved (*n* = 7). The fourth theme was living well, or ‘for the day’ (*n* = 24).

A significantly higher proportion of respondents aged 45 to 74 (78%) than over the age of 75 (46%) and under the age of 45 (67%) indicated intention take action due to an Awareness-raising presentation (Chi-squared test *P* = < 0.001). For ‘How to’ workshops, a significantly higher proportion of under 75 s compared with over 75 s (82% v 40%) indicated intention to take action due (*P* = 0.007), with no significant difference between those aged under 45 and those aged 45 to 74.

For both event types combined, respondents who had not previously discussed their own end of life preferences, compared with those who had, more often stated that they intended to take some action due to the event (67% v 79%, *P* = 0.001; and 69% v 79%, *P* = 0.011).

#### Follow-up

##### Response rate and potential bias

Of the 141 individuals who returned follow-up questionnaires, 100 had attended an ‘Awareness-raising’ presentation; 41 had attended a ‘How to’ workshop; and 15 indicated they had attended both types of event. The median time between baseline and follow-up was 13 weeks six days (range 11 weeks 3 days to 30 weeks 2 days).

Responders to both the baseline and follow-up surveys differed significantly from those who completed only the baseline survey. At baseline, those who went on to complete the follow-up survey had more often talked to somebody close to them about their own or the other person’s end of life and final wishes, and reported being more comfortable with these conversations (Table 4). Immediately post-intervention, follow-up responders had rated events as more relevant (chi-square test for trend *p* = 0.001) and more often indicated that they planned to take some action due to the event (118/141 (84%) v 245/357 (69%), *P* = 0.001). There were no significant differences between follow-up responders and non-responders in sex, neighbourhood deprivation or type of event.

**Table 4:** Percentage of baseline survey responders, by whether or not they responded to the follow-up survey, who reported having had different end of life conversations a baseline, and mean difference, on a scale of 1 to 10, in how comfortable they reported feeling about such conversations

	Had the conversation			Mean difference in how comfortable	
Topic of Conversation	Follow-up Responder	Follow-up Non-responder	*P*-value	Responder/non responder	*P*-value
Own end of life care	85/140 (61%)	167/355 (47%)	0.006	0.642	0.001
Own wishes for after your death	96/140 (69%)	189/349 (54%)	0.003	0.613	0.001
Another person’s end of life care	97/141 (69%)	166/351 (47%)	< 0.001	0.737	< 0.001
Another person’s wishes for after their death	94/140 (47%)	160/344 (67%)	< 0.001	0.648	0.002

##### Wills

Baseline and follow-up data on wills was available for 132 individuals. At baseline, 98 (74%) reported having a will, increasing to 100 (76%) at follow-up. The difference comprised two individuals who indicated they were thinking of making a will at baseline and had a will at follow-up, one of whom, elsewhere in the questionnaire, mentioned the intervention event as a motivating factor in writing a will. Of those without a will at baseline or follow-up, four who were not thinking of making a will at baseline were considering it at follow-up, while two who were considering writing a will were no longer considering it.

##### Talking about end of life preferences and bereavement

Between baseline and follow-up, there was a significant increase (9% respondents, *P* = 0.033) in the number of people reporting having ever talked to a close other about end of life preferences. There were no significant differences in numbers who had talked about another person’s end of life preferences, or supported/comforted somebody who had been bereaved (Table 5). Consistent with this, 12 respondents (9%) reported at baseline that they had never spoken about end of life preferences, then reported at follow-up that they had had those conversations due to the intervention.

**Table 5:** Number and percentage of follow-up survey respondents who indicated that they had talked to close friends and family about end of life wishes, or comforted somebody who had been bereaved, at baseline and follow-up, and change between the two time points

	Before			Follow-up			Difference			*P* value
	Awareness	How to	Total	Awareness	How to	Total	Awareness	How to	Total	Total
Own end of life care	59/99 (60%)	26/40 (65%)	85 (61%)	70/99 (71%)	27/40 (68%)	97 (70%)	11%	3%	9%	0.033
Own wishes for after your death	70/99 (71%)	26/40 (64%)	96 (69%)	79/99 (80%)	29/40 (73%)	108 (78%)	9%	9%	9%	0.033
Other’s wishes for end of life care	66/100 (66%)	29/39 (74%)	95 (68%)	68/100 (68%)	27/39 (69%)	95 (68%)	2%	−5%	0%	N/A
Other’s wishes for after their death	67/98 (68%)	24/39 (85%)	91 (66%)	67/98 (68%)	26/39 (74%)	93 (68%)	0%	2%	2%	0.44 (NS)
To comfort somebody who is bereaved	86/100 (86%)	36/39 (92%)	122 (88%)	83/100 (83%)	34/39 (87%)	118 (85%)	−3%	−5%	−3%	N/A

There were no differences between baseline and follow-up in how comfortable respondents reported being talking to close friends and family about end of life preferences or supporting somebody who had been bereaved (mean score for all five survey items combined 8.58 (95% CI 8.34 to 8.82) at baseline; 8.48 (8.23 to 8.74 at follow-up). Most respondents had similar scores at both time points; 108 (77%) were within one point of each other. To control for floor and ceiling effects, a further analysis was undertaken excluding individuals with a baseline score of one or ten. This yielded a small positive mean difference for each survey item, which reached significance for talking about own end of life care (Table 6). There were no apparent differences by type of event attended.

**Table 6:** Mean differences in baseline and follow-up scores for how comfortable respondents indicated that they would be talking about different about end of life wishes and bereavement, excluding individuals with baseline scores of one or ten

Topic of conversation	Sample size	Mean difference	*P*-value
Own end of life care	67	0.627	0.003
Own wishes for after your death	69	0.420	NS
Other’s wishes for end of life care	77	0.182	NS
Other’s wishes for after their death	77	0.052	NS
To comfort somebody who is bereaved	86	0.070	NS

##### Actions taken and changes made as a result of attending the event

Eighty respondents (64%) reported taking action due to the intervention. This included 58 (43%) who had talked about their own end of life preferences, 52 (39%) who had taken other action, and 30 (23%) who had both talked about their own end of life wishes and taken other action.

Immediately post-intervention 114 (84%) had indicated their intention to take some action; at follow-up 73 (64%) of these had taken action. In addition, 7/19 (37%) of those who indicated no intention to take action post-intervention reported they had taken action. Taking into account respondents whose reported intentions and actions were completely different (for example, intended to have a conversation but actually planned a funeral); only 43% of reported intentions and actions corresponded or overlapped.

In relation to types of actions taken, a fifth theme, ‘passing on the message’ emerged. This included four respondents who had arranged a similar or related information event, and two who had discussed arranging an event. Seven reported encouraging others to talk more or prepare better for death.

## Discussion

### Summary of findings

#### Characteristics of participants

Respondents were older than the UK population as a whole (46% were over the age of 65 compared with 22% of adults over 20 in England [26]); were more often female (76% compared with approximately 51%); and experienced lower levels of neighbourhood deprivation. The preponderance of females mirrors the experience of a project in Ireland, which used ‘Cafe Conversation’ events to stimulate discussion about similar issues [27]. This suggests that these type of events might be of more interest to, or more accessible by, women compared with men. The lower levels of deprivation are mainly a reflection of the relatively affluent area in which the intervention was conducted.

The observed trends for the proportion of people with a will to increase with increasing age and decreasing neighbourhood deprivation mirror those found in national surveys [15–17], although in age groups over the age of 45, the proportion with a will was slightly higher in this study, perhaps reflecting the lower levels of deprivation in the study area.

Also in line with national survey results, most respondents reported being comfortable talking about death, and older respondents reported being more comfortable than younger respondents. Both selection bias and response bias are possible, as those who were less comfortable talking about death may have been less likely to attend events and to complete the survey than those who were more comfortable. However, response rates to the baseline survey were high (74%), and over half the events were delivered to established groups as one of their regular meetings, with organisers reporting only a few members choosing not to attend. This provides further evidence against the common description of death as ‘taboo’ as a subject of conversation [28]. In a Canadian population survey [29, 30], only 9% agreed that ‘end of life is too sensitive a topic to talk about’.

The proportion of respondents reporting talking to somebody close to them about what they wished to happen after their death was also very similar, by age group, to the proportion in a national survey who reporting telling another whether they wished to be cremated or buried [15]. Neither experience of talking about end of life wishes or bereavement, or how comfortable persons reported being about these conversations, appeared to differ by sex or neighbourhood deprivation.

#### Effectiveness of the interventions

Both types of intervention event were well-received and rated as highly relevant by those who attended them. The relevance of the subject matter reflects the findings of a 2005 survey of people over the age of 55 [31], where most agreed with the statement: “I wish that death and dying were more openly discussed within society”. It also relates to the perceived high quality of the intervention; comments received indicated that the ideas presented, humour, sensitive facilitation and opportunities for discussion were particularly appreciated. These findings confirm the conclusions of an earlier systematic review [20] that, in the right context, people often appreciate the opportunity to talk about issues relating to death and end of life.

In published literature, other interventions that were well-regarded by participants, and were able to generate discussion, include a collaboration between academics and older people to develop an information booklet and peer education programme [32]; a series of public information ‘roadshows’ held in town centres [33] and publicly advertised ‘Café Conversation’ events [27]. All of these interventions, like the CLWDW events, were participatory or interactive in nature, covered subjects of universal relevance (such as planning funerals and coping with bereavement), and ensured that participation was both informed and optional. In contrast, an information-based end of life planning module, included without prior warning within an ‘expert patient’ chronic disease management education programme, was felt by participants to be inappropriate in that context, and caused distress to some who had recently been bereaved [34].

Of those who responded to all three parts of the survey, 84% indicated their intention to take action post-intervention, and 64% reported taking action at three month’s follow-up; the most frequent type of action being specific conversations with family members. These very high figures may partly reflect the observed response biases. To test the possible extent of this, we conducted a sensitivity analysis, assuming that no attendees other than those who responded to the 3-month follow-up survey and reported taking action were influenced by the event. This suggests that a minimum of 12% (141/676) attendees took action; still a significant proportion.

There was little difference between baseline and follow-up in how comfortable respondents reported being talking about end of life preferences and bereavement. This suggests that the positive impact on behaviour was not mediated by changes in confidence or attitude. In free-text comments, participants often described being ‘prompted’ to act, or realising that they needed to take action. This is consistent with the findings of UK population surveys, where the most common reason people give for not discussing end of life preferences is not that they feel uncomfortable about it, but that death seems a long way off [16, 18].

The interventions appeared to have limited success in encouraging people to make a will; only two follow-up respondents reported making a will between baseline and follow-up. This suggests the process of thinking about and writing a will might often take longer than three months, or perhaps that different, more practical interventions will be required to significantly increase will-making.

Respondents aged 45 to 74 rated events as more relevant than older and younger groups, and more often reported taking action as a result of attending events. It may be that the content was inherently more relevant to people within this age range, for example they may consider planning for end of life to be something relevant to their life-stage, but not something they had yet done anything about. It could also be that the content, style and delivery of the events was most suited to this age group. The CLWDW team are continuing to develop alternative interventions targeted towards other demographic groups, for example, theatre workshops for young students and ‘wills workshops’ in workplaces.

The positive impact of the CLWDW interventions could reach beyond those actually attending events. By encouraging discussion with others, the intervention could be acting as a ‘seed’ spreading ideas within the population. It has also inspired other similar events and projects, delivered independently of CLWDW, therefore acting as a wider community development initiative, which could make it more responsive and sustainable in the long term.

### Strengths and limitations of the study

The strength of the study was conducted in a ‘real life’ setting among males and females of different ages and backgrounds residing in both rural and urban areas. It included a follow-up phase, so that impact on actual behaviour, rather than just short term intentions, could be evaluated.

The main limitation was the unavoidable potential for non-response bias. This probably caused effect of the intervention on behaviour to be over-estimated, but did not change our conclusion that they were successful in meeting their objectives. The survey might also have acted as an intervention itself, prompting more thought on preparations for end of life. However, this is probably not a major methodological flaw as, in practice, questionnaires are often used immediately after events to both evaluate the event and intentionally prompt further reflection.

## Conclusions

As far as we are aware, this is the first follow-up study of a community intervention intended to improve upstream communication of end of life preferences and to normalise death as a subject of conversation.

The findings suggest that well-designed awareness-raising and educational events, delivered in a sensitive manner, can prompt the public to consider end of life preferences and discuss these with the people closest to them, and may increase recipient’s confidence in having those conversations. These types of intervention have the potential to reach beyond the immediate recipients, as attendees talk to others about issues, or even host their own similar events. The events evaluated in this study appeared to be relevant and effective for all age groups, but were most relevant and effective for those aged 45 to 74. Further research might identify interventions more suitable for younger and older age groups, and those who would not be reached by events like these.

## Additional files

Additional file 1: Final questionnaire part 1 and 2 awareness. Baseline’ and ‘post’ questionnaire for awareness-raising events. Copy of baseline and post-event questionnaires used at Living Well Dying Well ‘Awareness-raising’ events. (DOCX 33 kb)

Additional file 2: Final questionnaire part 1 and 2 how to Baseline’ and ‘post’ questionnaire for ‘how to’ events. Copy of baseline and post-event questionnaires used at Living Well Dying Well ‘How to’ events. (DOCX 35 kb)

Additional file 3: Final questionnaire part 3. Follow-up’ questionnaires for all event types. Copy of follow-up questionnaires used three months after Living Well Dying Well ‘Awareness-raising’ and ‘How to’ events. (DOCX 28 kb)
