# Supplementary material for: Discussing end of life wishes – the impact of community interventions?
Source: BMC Palliat Care. 2019 Mar 7;18:26. doi: 10.1186/s12904-019-0407-8 (PMC6407206; doi:10.1186/s12904-019-0407-8)
Supplement: Supplementary file 1 — Final questionnaire part 1 and 2 awareness. Baseline’ and ‘post’ questionnaire for awareness-raising events. Copy of baseline and post-event questionnaires used at Living Well Dying Well ‘Awareness-raising’ events. (DOCX 33 kb) [file 12904_2019_407_MOESM1_ESM.docx]

**Research Questionnaire**

**Talking about Death, Dying and loss**

We would be very grateful if you could take a few minutes to complete this short questionnaire to help us gain a better understanding of this very important subject area.

Your answers will be used as part of a research project conducted by the University of Liverpool about how people talk to those close to them about issues relating to dying, death and bereavement. The project is supported and funded by Cheshire Living Well Dying Well Public Health Programme which is working locally to help raise awareness of the benefits of openly discussing these issues. Your answers could guide us in developing ways to help make sometimes difficult conversations easier and more comfortable for people.

The completed questionnaires will be kept securely at the University of Liverpool and any information you give will be kept confidential.

We would like you to include your contact details as we are interested to keep in touch and possibly ask you to complete a further questionnaire. However, you do not have to give us your contact details if you do not want to.

**Miss Katharine Abba**

**PhD student**

**Prof Mari Lloyd-Williams**

**Supervisor**

| **Part 1**  Type of Event: **Introductory session** Date:   1. **ABOUT YOU**   ***1. Are you male or female? (tick one)***  □ Male □ Female |
| --- |
| \| □ Under 25  □ 55 to 64 \| □ 25 to 34  □ 65 to 74 \| □ 35 to 44  □ 75 to 84 \| □ 45 to 54  □ 85+ \|  \| \| --- \| --- \| --- \| --- \| --- \| |

***2. How old are you? (tick one)***

***3. Who do you live with? (tick as many as apply)***

| □ Husband, wife or partner  □ Friends  □ Other (please state).................................... | □ Other family  □ I live alone |
| --- | --- |

***4. What do you do? (tick any that apply)***

| □ Work full-time  □ Work part-time  □ Not working due to sickness or disability  □ Unemployed | □ Full-time carer  □ Full-time home-maker  □ Retired  □ Other (please state)........................................ |
| --- | --- |

***5. What town or village do you live in?*** ......................................................................

***6. What is your postcode?* ...................................................***

**We will use your postcode to see what type of area you live in, for example, town or countryside.*

***7. Are you considering making a will?***

□ Yes □ No □ I have already made a will

□ I already have a will but am thinking of making changes to it

1. **TALKING ABOUT *YOUR* WISHES**

***8. Have you ever talked with close family or friends about your wishes about your care if you became unwell and at the end of your life?***

**□** Yes

□ No

***9. How comfortable would you feel about talking with a close family member or friend about your wishes about your care if you became unwell and at the end of your life, if you wanted to talk about it?***

(circle one answer on a scale of 1 to 10)

*1= Not at all comfortable 10= Completely comfortable*

**1 2 3 4 5 6 7 8 9 10**

***10. Have you ever talked with close family or friends about your wishes about what you would like to happen after your death?***

**□** Yes

□ No

***11. How comfortable would you feel about talking with a close family member or friend about your wishes about what you would like to happen after your death, if you wanted to talk about it?***

(circle one answer on a scale of 1 to 10)

*1= Not at all comfortable 10= Completely comfortable*

**1 2 3 4 5 6 7 8 9 10**

1. **TALKING ABOUT *OTHER PEOPLE’S* WISHES**

***12. Have you ever talked with close family or friends about their wishes about their care if they became unwell and at the end of their life?***

**□** Yes

□ No

***13. How comfortable would you feel about talking with a close family member or friend about their wishes about their care if they become unwell and at the end of their life, if they wanted to talk about it?***

(circle one answer on a scale of 1 to 10)

*1= Not at all comfortable 10= Completely comfortable*

**1 2 3 4 5 6 7 8 9 10**

***14. Have you ever talked with close family or friends about their wishes about what they would like to happen after their death?***

**□** Yes

□ No

***15. How comfortable would you feel about talking with a close family member or friend about their wishes about what they would like to happen after their death, if they wanted to talk about it?***

(circle one answer on a scale of 1 to 10)

*1= Not at all comfortable 10= Completely comfortable*

**1 2 3 4 5 6 7 8 9 10**

1. **TALKING ABOUT *BEREAVEMENT***

***16. Have you ever needed to comfort or support a friend or family member who has recently experienced the death of somebody close to them or is caring for somebody who is dying?***

**□** Yes

□ No

***17. How comfortable would you feel having a conversation with a family member or friend about the death of somebody close to them, if they wanted to talk about it?***

(circle one answer on a scale of 1 to 10)

*1= Not at all comfortable 10=Completely comfortable*

**1 2 3 4 5 6 7 8 9 10**

**___________________________________________________________________________**

**Thank you very much for your help**

**We have a few more questions that we would like to ask you, but not until after the session.**

**Please keep this questionnaire with you until then.**

|  |  |
| --- | --- |

**Part 2**

***1. How relevant was the presentation to you and your life?*** (circle one answer on a scale of 1 to 5)

*1= Not at all relevant 5= Extremely relevant*

**1 2 3 4 5**

| ***2. Was anything at the presentation particularly relevant, useful or thought-provoking for you?***  □ Yes  □ No  If ‘yes’ what was it?  ***5. Are you planning to have any specific conversations with family or friends because of anything you have heard today?***  □ Yes  □ No  If ‘yes’ could you describe?  ***4. Are you now considering making a will?***  □ Yes □ No □ I have already made a will  □ I already have a will but am thinking of making changes to it |
| --- |
| ***3. Did the presentation inspire you to do anything else or make any other changes in your life?***  □ Yes  □ No  If ‘yes’ what were they? |

**Permission to contact you for further research**

This questionnaire survey is part of a larger research project.

We would like as many people as possible to complete a similar questionnaire in about three months’ time to help us to determine whether work that is being done by the Cheshire Living Well Dying Well Partnership makes any difference to people.

We are also looking for about 30 people to talk to us in more depth about their views about the importance (or not) of talking about end of life, death and bereavement, and what they think might help people to have these conversations if they want to.

We would like your permission for us to contact you about either of these additional research activities. We will give you more information about this research at the time, and you will be free to choose whether or not to participate.

**I give my permission for a researcher to contact me about me about further research in people’s views and experiences of talking about end of life, death and bereavement.**

□ Yes □ No

**Name:**

**Address:**

**Email:**

**Telephone: Mobile:**
