# Supplementary material for: Discussing end of life wishes – the impact of community interventions?
Source: BMC Palliat Care. 2019 Mar 7;18:26. doi: 10.1186/s12904-019-0407-8 (PMC6407206; doi:10.1186/s12904-019-0407-8)
Supplement: Supplementary file 3 — Final questionnaire part 3. Follow-up’ questionnaires for all event types. Copy of follow-up questionnaires used three months after Living Well Dying Well ‘Awareness-raising’ and ‘How to’ events. (DOCX 28 kb) [file 12904_2019_407_MOESM3_ESM.docx]

**Research Questionnaire**

**Talking about death, dying and loss**

We would be very grateful if you could take a few minutes to complete this short questionnaire. It is a follow-up to the questionnaire you completed around the time of the Living Well Dying Well Public Health Programme presentation at St Luke’s Hospice. Some of the questions are very similar to those we asked before; this is done on purpose to see whether and how things have changed.

Your answers and those of other people are very important to us and will be used to see whether these sessions make any difference to the people who attend, and how. Please be honest in your responses, there is no need to be polite.

The completed questionnaires will be kept securely at the University of Liverpool and any information you give will be kept confidential.

It is up to you whether you complete the questionnaire or not, and you may also choose to leave out any questions that you do not want to answer.

**Miss Katharine Abba**

**PhD student**

**Prof Mari Lloyd-Williams**

**Supervisor**

| Please write today’s date here: __________________________   1. **ABOUT YOU**   ***1. Are you considering making a will?***  □ Yes □ No □ I have already made a will  □ I already have a will but am considering making changes to it  ***2. Have you ever talked with close family or friends about your wishes about your care if you became unwell and at the end of your life?***  **□** Yes  □ No  ***3. How comfortable would you feel about talking with a close family member or friend about your wishes about your care if you become unwell and at the end of your life, if you wanted to talk about it?***  (circle one answer on a scale of 1 to 10)    *1= Not at all comfortable 10= Completely comfortable*  **1 2 3 4 5 6 7 8 9 10** |
| --- |
| ***4. Have you ever talked with close family or friends about your wishes about what you would like to happen after your death?***  **□** Yes  □ No  ***5. How comfortable would you feel about talking with a close family member or friend about your wishes about what you would like to happen after your death, if you wanted to talk about it?***  (circle one answer on a scale of 1 to 10)    *1= Not at all comfortable 10= Completely comfortable*  **1 2 3 4 5 6 7 8 9 10**  ***6. Have you ever talked with close family or friends about their wishes about their care if they became unwell and at the end of their life?***  **□** Yes  □ No  ***7. How comfortable would you feel about talking with a close family member or friend about their wishes about their care if they become unwell and at the end of their life, if they wanted to talk about it?***  (circle one answer on a scale of 1 to 10)    *1= Not at all comfortable 10= Completely comfortable*  **1 2 3 4 5 6 7 8 9 10** |

***8. Have you ever talked with close family or friends about their wishes about what they would like to happen after their death?***

**□** Yes

□ No

***9. How comfortable would you feel about talking with a close family member or friend about their wishes about what they would like to happen after their death, if they wanted to talk about it?***

(circle one answer on a scale of 1 to 10)

*1= Not at all comfortable 10= Completely comfortable*

**1 2 3 4 5 6 7 8 9 10**

***10. Have you ever needed to comfort or support a friend or family member who has recently experienced the death of somebody close to them or is caring for somebody who is dying?***

**□** Yes

□ No

***11. How comfortable would you feel talking with a family member or friend about the death of somebody close to them, if they wanted to talk about it?***

(circle one answer on a scale of 1 to 10)

*1= Not at all comfortable 10=Completely comfortable*

**1 2 3 4 5 6 7 8 9 10**

1. **ABOUT THE LIVING WELL DYING WELL PRESENTATION AND ‘HOW TO’ WORKSHOP**

| ***12. Which events did you attend?***    □ An hour-long presentation by the ‘Living Well Dying Well’ team  □ A two or three hour long ‘How to’ workshop  □ Both a presentation and the workshop |
| --- |

| ***13. Was anything at the presentation or the ‘how to’ workshop particularly relevant, useful or thought-provoking for you?***  □ Yes  □ No  If ‘yes’ what was it? |
| --- |

| ***14. Since the presentation or ‘how to’ workshop, have you talked with anybody close to you about your own end of life wishes?***  □ Yes  □ No  ***If ‘yes’ do you think the attending the presentation or workshop encouraged you to do this?***  □ Yes  □ No, I would have done this anyway    ***15. Since the presentation or workshop, have you done anything else or made any other changes in your life because of what you heard there?***  □ Yes  □ No  If ‘yes’ what were they? |
| --- |

|  |
| --- |

***16. Do you have any other comments?***

Thank you very much for your help.

Please return the completed questionnaire using the enclosed pre-paid envelope to:

**Miss Katharine Abba**

**Academic Palliative and Supportive Care Studies Group (APSCSG)**

**Waterhouse Building**

**Block B 1st Floor**

**1-5 Brownlow Street**

**Liverpool**

**L69 3GL**

|  |
| --- |
